# A qualitative study exploring how an Emergency Medicine leadership programme was received in Ghana

**DOI:** 10.1016/j.afjem.2026.100993

**Published:** 2026-08-08

**Authors:** Alexandra Taylor, Shivani Swina Patel, Emmanuel Acheampong, Richard Parris, Nikki Biggs, Kwaku Nyame, Anisa Jabeen Nasir Jafar

**Affiliations:** a University Hospitals Bristol & Weston NHS Foundation Trust, Bristol, United Kingdom; b Northern Care Alliance NHS Trust, Greater Manchester, United Kingdom; c Komfo Anokye Teaching Hospital, Kumasi, Ghana; d Bolton NHS Foundation Trust, Bolton, United Kingdom; e Frimley Health NHS Foundation Trust, Surrey, United Kingdom; f The University of Manchester, Humanitarian and Conflict Response Institute, Manchester, United Kingdom; g Emergency Department, Salford Royal Hospital, Northern Care Alliance NHS Foundation Trust, Greater Manchester, UK

**Keywords:** Leadership, Education, Emergency medicine, Qualitative research

## Abstract

**Introduction:**

Emergency Medicine (EM) is a developing specialty in several West African countries, where practitioners are increasingly assuming leadership roles despite limited training. The EM Leaders programme, originally designed for the UK context, offers leadership training tailored to EM. This study explores the reception, relevance, and adaptability of the EM Leaders programme when delivered in the Ghanaian EM context.

**Methods:**

This qualitative study used focus groups conducted before and after the EM Leaders programme in Ghana, to explore participants’ experiences. Delegates in the programme were EM practitioners from Ghana (and two from Nigeria). The research followed a phenomenological approach with reflexive thematic analysis. Transcripts were analysed, audio data analysis was socio-culturally informed, and findings were triangulated with participants.

**Results:**

Seven delegates took part as research participants. Key motivations for leadership learning included the rapid growth of EM as a specialty and early exposure to leadership roles without formal training and mentorship. The programme broadened participants' understanding of leadership beyond management to include teamwork, delegation, problem-solving, and self-awareness. Participants expressed a strong desire to formalise leadership training into local EM curricula. Contextual differences such as language, socio-cultural norms around hierarchy, and feedback practices were highlighted. Adaptations including socio-culturally sensitive examples, slower delivery, and diasporan engagement were suggested to optimise the programme’s relevance. The programme inspired participants to explore strategies for embedding leadership skills into their systems, despite challenges with human and financial resources.

**Conclusion:**

The EM Leaders programme was positively received and demonstrated potential for applicability within the Ghanaian EM context. Incorporating additional management content, mentorship development, and socio-cultural tailoring could enhance its relevance and impact. Participants endorsed embedding formal leadership training within EM education, acknowledging its importance in an evolving healthcare landscape. This study suggests early signals for the programme’s transferability, and yet underscores the necessity of contextualising leadership development to advance global EM development.

## African relevance

• Development and implementation of intervention took place in Ghana.
• Participants were mainly Ghanaian EM practitioners, with some from Nigeria.
• Strengthens African research on delivering EM-focused leadership training.
• Highlights the need for socio-cultural adaptation of imported training tools.

## Introduction

### Background of EM development

Emergency medicine (EM) in Ghana is a new and rapidly growing specialty [1]. With formal EM training first established in 2009 [2], it joins a growing number of West African countries in the process of developing the speciality. Nearby in Nigeria, the first specialty primary examinations were held in 2021 [3,4]. Both countries are at different stages of building formal emergency care systems.

The Ghanaian College of Physicians and Surgeons (GCPS) has sought external support from international EM colleagues, working in other healthcare systems, familiar with the challenges of leading and growing EM as a specialty. It was concurrently noted that three key modules of the UK EM Leaders programme aligned with the GCPS priority areas [5]. The Ghanaian faculty is small in comparison to other, more established colleges and the leadership demands asked of it is great. There are currently 12 Ghanaian EM clinicians senior enough to train residents in three active training centres with approximately 150 residents. Numbers are growing which creates significant pressure on the small numbers of senior and experienced trainers.

Leadership skills have been acquired experientially at a relatively early career-stage and have rarely been formally taught; a training gap noted also in other sub-Saharan African studies [[6], [7], [8], [9]]. A specific ask of the Royal College of Emergency Medicine (RCEM), in the context of an established partnership since 2021, was to support the development of individual, systemic, clinical, and educational leadership skills in Ghana which are required at a local, regional, and national level [10]. Whilst much more in its infancy, the partnership developing between RCEM and the National Postgraduate Medical College of Nigeria, has evolved some similar development requests hence the decision to include colleagues from this West African country.

Good medical leadership is associated with high-quality patient care [11,12] and is a specific skill that can and should be taught and embedded at an early stage of medical training [13,14]. Many leadership programmes are generic in nature and borrow theory and practice from non-medical contexts [12]. Given the unique environment and skill set EM demands, the suitability of such courses has been challenged [15]. The EM Leaders programme for EM trainees was developed by the RCEM in conjunction with the Faculty for Medical Leadership and Management and NHS England (at the time, known as Health Education England) [10]. The programme was introduced in 2018 to support high quality patient care and more specifically, to address burnout and retention within the speciality. Recent publications evaluating the programme confirmed the continued need for specific EM leadership training and highlighted the quality of the programme [11,16].

The EM Leaders programme was delivered in Ghana through an RCEM–GCPS partnership (funded by Global Health Partnerships (formerly Tropical health and Education Trust (THET)) as part of the Global Health Workforce Programme funded by the UK Department for Health and Social Care) to advance postgraduate emergency medical education [17]. In April 2024, an RCEM team was invited by Ghanaian partners to support leadership development within the Ghanaian EM Faculty, using the core modules of the EM Leaders programme, providing an opportunity to assess the programme’s generalisability beyond the UK.

## Methods

### Study design

A majority EM practitioner research team with support from RCEM undertook this qualitative study utilising focus groups. Guided by a contextualist epistemology, the study employed a phenomenological approach to understand participants' lived experiences, and utilised reflexive thematic analysis. Reporting adheres to the Standards for Reporting Qualitative Research checklist [18]. The research focus was identified following a discussion between RP and AJ expressing a desire to understand if the course translated into a Ghanaian EM setting and met the expectations of the delegates. In advance of the leadership programme, all delegates on the programme were provided with a summary of the planned study.

Focus groups were conducted pre- and immediately post- the EM Leaders programme by four UK researchers familiar with the EM Leaders programme (but with no conflict of interest as to the format in which it is delivered). Focus group questions can be found in online appendix and targeted parts *‘a) participants' reactions to training’* and *‘b) participants’ learning as a result of training*’ of the Kirkpatrick framework for assessing a training programme [19].

The study was deemed not to require formal ethics approval by the University of Manchester’s Ethics Decision Tool. In addition, Kwame Nkrumah University of Science and Technology (KNUST) confirmed no requirement for ethical approval.

There are a limited number of Emergency Physicians in Ghana, at the time there were eight EM senior clinician trainers in the country, all of whom were invited by the GCPS, as well as several other key senior national EM leaders, and then the invitation was extended to include Nigerian participants who were put forward by the West African College of Physicians (WACP) due to a keen interest in developing the specialty of EM.

### Data collection

UK trainers delivered the course in April 2024. Participants were consented for the research, and focus groups were conducted before and after the course using a topic guide developed by AJ and RP (see e-Extra Content online). Each focus group was chaired by two faculty members. Participation was emphasised as being voluntary and separate conceptually from the course itself. Assurances were reiterated with respect to anonymity such that any manuscript would be presented to all participants prior to publication. Focus groups lasted approximately 1 hour each and were carried out in English within the setting of the leadership course provision at the GCPS central office/campus. Discussions were recorded with a digital voice recorder, though one pre-course focus group failed to record; field notes were taken instead. Recordings were then professionally transcribed.

### Positionality

The faculty members who led the focus groups comprised three UK EM practitioners and one member of the RCEM international team. They have partaken in the UK partnership with Ghana over several years and have established working relationships with many of the focus group participants. To a lesser extent the same is true of the Nigerian participants.

### Analysis

Analysis of anonymised transcripts followed Braun and Clarke’s six-step reflexive thematic analysis (Fig. 1) [20]. Independent researchers AT and SP, blinded to the EM Leaders content, conducted inductive coding in Microsoft Word. First-order codes were generated separately, then collated and refined into higher-order sub-themes through iterative discussion. To enhance contextual validity, EA, with knowledge of the Ghanaian context, reviewed audio recordings and extracted additional codes, including implicit meanings. These were integrated with AT/SP’s findings, and final themes were identified, defined, and named. The field Notes from the unrecorded pre-course focus group were not used for coding but later confirmed for alignment with the developed themes. A master list of themes and sub-themes was then reviewed in a triangulation meeting with four focus group participants, leading to minor refinements.

## Results

In total seven people participated in leadership training, five Ghanaian EM consultants (including two senior national Ghanaian EM leaders) and two Nigerian EM consultants. All those who attended the leadership training agreed to participate in the focus groups however one participant could not attend the pre-course focus group, and one different invitee was able to attend only the post-course focus group, due to prior time commitments (Table 1).

**Table 1:** Participants within focus groups, role-breadth, practising countries, number of years post-graduate clinically.

	Participants	Countries of practice	Role-breadth	Number of years post-graduate to the nearest 5 years
Pre-course	A,B,C,E,F,G	Ghana x 4 Nigeria x 2	• All consultants • 2 heads of department • 2 senior roles with in national clinical training college	15 20 20 20 25 35
				
Post-course	A,B,D,E,F,G	Ghana x 4 Nigeria x 2	• All consultants • 2 heads of department • 2 senior roles with in national clinical training college	15 20 20 20 25 35

Four focus groups were conducted (two pre-course, two post-course) with seven participants in total. Themes and outcomes of the focus groups are shown in Table 2, Table 3, Table 4, Table 5.

**Table 2:** Themes and subthemes identified.

**Theme**	**Sub-themes**
Motivations for seeking leadership learning	a) EM as a junior and growing specialty b) External pressure to lead despite apprehension. c) Recognition of lack of training and mentorship
Ideas of leadership: management vs leadership skills	a) Evolving perspectives on leadership – problem-solving, teamwork, delegation, and self-awareness b) Expression of interest for support in managerial functions
Formalising leadership into curriculum	a) Desire to formalise leadership training. b) Maintaining currency and empowerment c) Troubleshooting strategies for leadership introduction
Contextual differences	a) Language choice b) Ability to speak out – courageous conversations. c) Hierarchy d) Fluidity of emergency medicine (EM) and culture

**Table 3:** Non-technical skills identified by delegates.

Problem solving and teamwork	*“I've learned a new way of solving problems [through] teamwork”* *“I discovered that your problem will be at the weakest link, not the strongest’’*
Delegation	*‘’[The] job of a good leader to know when to step aside and let somebody lead’’* *“It’s reshaped my thinking…leadership means everybody is also leading [through] delegation…the leader will have to set an example by supporting that person’’*
Self-awareness	*‘’ [I found it relevant] evaluating yourself, knowing what kind of a leader you are… to improve the team’’* *‘’ [I learned] … its [important] to know what is going to work for individuals you are dealing with and the setting which you may find yourself.’’*

**Table 4:** Identified strategies for leadership introduction.

Incentivising	*“We may have to use non-cash methods to reward our trainers… like getting your article published”*
Maintaining course practicality	*“I’m really grateful for the lectures, the practicality of most of the sessions and for inspiring me to be a better me in my work, and also putting that interest, reigniting that interest to also look for others and guide them in their work and their training.”*
Time and resource availability	*“We just need to get the resources and make it available… in our department, they have one day already as [an] academic day.”*
Financial strategies	***“****we don’t have the funds to sort of physically give cash rewards to our trainers if they do the mentorship. We are still discussing how we can motivate trainers”*

**Table 5:** Suggestions to counteract translational barriers.

Panel of diasporan colleagues	*‘’[Having] a panel of diasporan colleagues to make suggestions for things to do as icebreakers/introductory things’’*
Analogies and proverbs	*‘’Finding out what is popularly known or relevant in Ghanian or Nigerian culture… [*e.g.*, Incorporating] Kwaku Ananse… proverbs and stories to illustrate’’*
Utilising technology	*“The facility to slow down to, you know, point five speed or point seven five speeds…so that they don’t feel they have to…go at full speed, ‘cause English is not their first language.”*

### Motivations for seeking leadership learning

#### EM as a junior and growing specialty

The youth and growth of EM in Ghana and Nigeria is driving a motivation and requirement to upskill in leadership to advance the specialty and build management systems.

*“Our trainees are moving out to be literally heads of institutions… very, very early,”*

#### External pressure to lead despite apprehension

Participants offered that they were taking leadership positions early in their EM careers, because of a lack of experienced candidates. They felt a pressure to do this even if they felt reluctant, under qualified or unprepared for these roles.

*“When I became a Fellow, there was really nobody on the ground, so you were pushed into things”*

#### Recognition of lack of training and mentorship

Despite the need for leadership, participants felt there was a lack of training in leadership to adequately equip them. There were no leadership role models, mentors or financial resources to encourage learning leadership. This necessity, and frustration with current systems, had motivated them to seek leadership training.

*“Suddenly we had to take on these tasks without the requisite skills”*

### Ideas of leadership: management versus leadership skills

#### Evolving perspectives on leadership

One participant noted the programme “*solidified”* their understanding of leadership, broadening it beyond management to include non-technical skills. Another felt it provided a “*structure”* for addressing challenges in emergency medicine. Participants also recognised the importance of focusing on team needs to achieve the best outcomes.

#### Expression of interest for support in managerial skills

Participants felt management skills were essential prerequisites for effective leadership in their context, given the heavy management burden they carried. They felt future leadership courses should reflect this.

*‘’Leadership I think includes management as well… in terms of being the head, the administrative bit as a leader, how are you going to set goals for yourselves, how am I going to run my department…”*

*‘’Specific management things like [organizing] teaching, [maintaining] lines of communication to the chief exec… recruitment… how do you develop policy…’’*

### Formalising leadership into curriculum

#### Desire to formalise leadership training

While informal, intuitive learning was already taking place, participants felt formalising and standardising it would clarify role expectations and limitations. An overarching curriculum or programme could maximise learning, safeguard roles such as mentorship, and ensure leadership training is not overlooked.

*“Currently, we are using our intuition, and also the experience that we’ve got from our senior colleagues.”*

*“So, in a way you now begin to use the mentee as a domestic staff, trying to run errands… But you should know your role as a mentor… so bringing it into the curriculum would really help”*

#### Maintaining currency and empowerment

Following the course, participants felt empowered to approach challenges with new perspectives and strategies. They emphasised that leadership skills require ongoing refreshers to avoid knowledge fatigue and maintain currency.

*“It should probably be a bit regular; I mean, we keep updating ourselves, we keep reminding ourselves”*

#### Troubleshooting strategies for leadership introduction

There was a noticeable shift after the course with participants troubleshooting how best to introduce and incentivise leadership training. This gave the impression that the course had motivated and inspired participants and they wished to transfer these skills.

*“How do we step some of this down to the residents’ programmes, take one of the presentations there or if the department allow in the departmental programmes”*

In the subsequent triangulation one participant highlighted human resources as an obstacle:

*“we have a huge incapacitation here, in term of human resource, in order to do it effectively, and I think it was one of the challenges that we anticipated.”*

### Socio-cultural shifts and challenges

Participants noted that course scenarios were transferable across emergency medicine settings in the UK, Ghana, and Nigeria, but key contextual differences still needed consideration. For example, one stated:

*‘’I’m beginning to believe EM is the same everywhere… maybe we need to tweak the language and some small context… but the scenarios are the same’’*

#### Language choice

With regards to course structure, participants commented on difficulty in understanding accents, speed of speech, and a lack of transferability in some analogies and vocabulary.

*‘’English proverbs and sayings [are] not necessarily transferable’’*

*“In Ghana no one says 'bloke'’’*

#### Socio-cultural shifts

Participants observed a shift toward greater openness in communication, contrasting with traditional norms of silence and indirect feedback, particularly in Ghana. Initiatives like ‘*Courageous Conversations’* helped separate the issue from the individual, enabling constructive dialogue.

“People don’t speak up… people talk amongst themselves [rather than] confronting the person”

Hierarchical structures—both generational and professional—remained barriers to learning and voice. However, younger professionals were increasingly willing to question authority and embrace collaborative person-centred leadership.

*“The consultant may say something that is no longer current… nobody would talk”*

*“Some of the master trainers were nurses in my department and they were now training me … I think it’s got to do with the younger generation… we accept things more easily”*

*“We are now having people who question the system and with that comes the culture of speaking up…”*

As emergency medicine evolves in the region, participants described a willingness to learn from other systems and adapt practices locally, reinforcing a culture of openness, continual improvement, and creation of psychologically safe spaces.

*“We came here from Nigeria to see the structure of emergency medicine… trying to find out what works”*

## Discussion

The evolution of EM alongside the increased recognition of the importance of scaling up the speciality [1,3] creates both opportunities and pressures for practitioners to assume leadership roles as this training competes with so many other demands. Echoing studies in the US and low- and middle-income countries [6,7,21,22], our findings suggest that, in the absence of formal leadership training, skill development is often acquired informally through observation and experience. This reliance on ad-hoc learning contributes to uncertainty and apprehension regarding leadership capabilities. This supports findings of Colella et al. [7] endorsing the need for structured contextualised programmes in complex emergency medicine environments where no single leadership model is universally applicable [23,24].

Internationally, specialised EM leadership programmes, such as EM Leaders, have demonstrated potential to formalise development and broaden understanding beyond managerial skills [7,11]. Participants reported gains in non-technical competencies including delegation, self-awareness, teamwork, problem-solving, adaptive leadership, person-centred approaches, and fostering psychologically safe spaces. This is consistent with Knowles’ adult learning principles [25] and social learning theory underpinning EM Leaders [11].

Studies [22,26] acknowledge a clear definition of leadership in emergency medicine is lacking internationally, which may lead to variability in leadership practises and expectations. The participant-defined non-technical outcomes generate empirical insights that could contribute to international efforts to establish and clarify the definition of leadership within emergency medicine [23,24]. Moreover, participants expanded their concept of leadership to include mentoring, policy, and financial management, reflecting the need to address organisational and system-level factors in programmes [26]

Our findings align with Kneafsey et al. [11], emphasising the need for context-specific, adaptive leadership programmes to support EM development and cautioning against assumptions of homogeneity across sub-Saharan Africa. While EM Leaders was developed in the UK, participants noted substantial overlap with local leadership needs but stressed the importance of socio-cultural and contextual adaptation, thereby building on previous evaluations of sub-Saharan programmes [7]. Effective knowledge transfer requires careful attention to language, local proverbs, culturally sensitive references, and technology-enabled learning, as well as engagement with diaspora colleagues.

The programme also motivated participants to explore strategies for sharing and sustaining leadership learning, suggesting potential for ripple effects within local EM systems. However, as identified across the continent [6,27,28], systemic challenges, including human resource limitations, financial constraints, and limited time, may impede formal integration into curricula. Socio-cultural nuances, such as reluctance to voice concerns, further underscore the need for safe-space approaches and reflective mechanisms for effective programme delivery and evaluation.

The evaluation aligns with Kirkpatrick’s framework for training assessment, which includes: (a) participants’ reactions, (b) participants’ learning, (c) change in behaviour, and (d) organisational impact [19]. Our study primarily captures aspects (a) and (b) — participants’ experiences and perceived learning — while (c) and (d), representing behavioural change and system-level effects, remain avenues for future research. However, our study has provided a richness of data on the influence of context and culture not captured in Kirkpatrick’s framework and building on previous studies. A third focus group conducted with participants may offer further insight into real-world applicability and longevity of EM Leaders learning in these contexts.

## Limitations

The study was led through the lens of UK-based emergency medicine clinicians with specific positionality which may have impacted the direction of questioning. To mitigate some aspects of this, a third local Ghanaian researcher assisted with coding audio content, providing socio-cultural insights and framing. The participant number is low however as a proportion of Ghanaian EM the narrative is highly representative of senior leaders and therefore significant power rests within the data which achieved breadth and depth, especially by the nature of triangulation. Interviewers occasionally used compound or leading questions, potentially limiting responses. As acknowledged in a Ugandan study [7], social desirability may limit participant candour in cultures where voicing criticism is uncommon. The interviewed population was also small and cannot have represented the whole spectrum of relevant practitioners. Audio recordings were incomplete for one pre-course meeting, and replacement notes were therefore triangulated after secondary interpretation.

Additionally, this study represents a partial evaluation of the EM Leaders programme, capturing only participants’ reactions and learning (Kirkpatrick levels a and b) [19]. Behavioural changes and organisational impact (levels c and d) were not assessed. Socio-cultural differences and context-specific challenges further highlight the importance of tailored evaluation strategies, reflective diaries, and safe-space mechanisms for optimising rich data collection.

## Conclusion

The development of EM in Ghana and Nigeria offers a unique opportunity to consider how to embed leadership training within evolving emergency care systems. This momentum is not confined to a single region; it reflects a broader trend. Since delivery, the EM Leaders programme has been discussed internationally with the African Federation for Emergency Medicine, key GCPS EM faculty members, the National Postgraduate Medical College of Nigeria and the Ethiopian faculty for contextual adaptation and delivery.

EM Leaders broadens understanding of non-technical leadership skills and motivates participants to apply and share learning. To maximise impact, the programme should expand to include mentoring, management, and system-level leadership, while adapting delivery strategies, language, and cultural references to local contexts. Participants recognise systemic barriers but emphasise the programme’s necessity and potential, suggesting that contextually adapted, formalised leadership development is essential for the growth and professionalisation of EM in these settings.

Future focus groups conducted with participants after the passage of significant time since this study was conducted may offer further insight into real-world applicability and longevity of EM Leaders learning in these contexts - with specific focus on the behavioural change and organisational impact

## Dissemination of results

The findings of this research study were shared within the community, from which the data was collected through triangulation with the physicians who were interviewed. These discussions allowed for the exchange of insights, clarification of conclusions, and the opportunity to gather feedback directly from the healthcare professionals actively engaged with the research.

## Declaration of generative AI and AI-assisted technologies in the manuscript preparation process

During the preparation of this work the author(s) used chatGPT to suggest rewording to reduce word count and to check formatting of the references. After using this tool/service, the author(s) reviewed and edited the content as needed and take(s) full responsibility for the content of the published article.

## CRediT authorship contribution statement

**Alexandra Taylor:** Formal analysis, Writing – original draft, Writing – review & editing. **Shivani Swina Patel:** Formal analysis. **Emmanuel Acheampong:** Formal analysis. **Richard Parris:** Conceptualization, Funding acquisition, Investigation, Writing – original draft, Writing – review & editing. **Nikki Biggs:** Conceptualization, Funding acquisition, Investigation, Writing – original draft, Writing – review & editing. **Kwaku Nyame:** Writing – review & editing. **Anisa Jabeen Nasir Jafar:** Conceptualization, Funding acquisition, Writing – review & editing.

## Declaration of competing interest

This research was funded through the Global Health Partnership (formerly THET) as part of the Global Health Workforce Programme funded by the UK Department for Health and Social Care. The authors declared no further conflict of interest.

## Appendix Supplementary materials

Image, application 1

## Footnotes

Supplementary material associated with this article can be found, in the online version, at doi:10.1016/j.afjem.2026.100993.
