# Supplementary material for: A qualitative study exploring how an Emergency Medicine leadership programme was received in Ghana
Source: Afr J Emerg Med. 2026 Aug 8;16(3):100993. doi: 10.1016/j.afjem.2026.100993 (PMC13485627; doi:10.1016/j.afjem.2026.100993)
Supplement: Supplementary file 1 [file mmc1.docx]

**Appendix 1**

**Appendix 2**
**Focus Group Questions**

**Introduction:**

What the purpose of the focus group is.

How long the focus group will last and that it will be repeated after the course.

Ensure consent forms and participation sheets sorted.

Reminder that this will be recorded and anonymised.

**Warm up questions**

Tell me about yourself.

What is your background?

What are you most looking forward to?

Is there anything you are apprehensive about?

**Pre-course questions:**

What is the group interest in leadership?

How does leadership fit within their role/health system?

What is the expectation of what they will gain from the course?

Are there any specific leadership approaches/ideas/techniques which you are already familiar with?

What are the most important aspects of leadership that you want to develop/understand?

**Post-course questions:**

How did the course fit with expectations?

How relevant was the course content to their role/healthcare system?

Has anything changed in their perspective of leadership?

If they were to attend the same course, what would they change?

Did anything really stand out as not fitting their context?

**Appendix 3
Figure 1**: Six Phases of Reflexive Thematic Analysis – adapted from Braun and Clarke (20)
